# Male Nursing Students’ Perception of Gender Barriers in Nursing Curricula in an Iranian University of Medical Sciences

**DOI:** 10.17533/udea.iee.v40n1e03

**Published:** 2022-03-29

**Authors:** Fahimeh Alsadat Hosseini, Kobra Parvan, Maryam Shaygan, Brian Thomson

**Affiliations:** 1 Nurse, Ph.D. Shiraz University of Medical Sciences, Shiraz, Iran. Email: fhoseini221@gmail.com Shiraz University of Medical Sciences Shiraz Iran fhoseini221@gmail.com; 2 Nurse, M.Sc. Tabriz University of Medical Sciences, Tabriz, Iran. E mail: parvank@yahoo.com. Corresponding author. Tabriz University of Medical Sciences Tabriz University of Medical Sciences Tabriz Iran parvank@yahoo.com; 3 Nurse, Ph.D. Shiraz University of Medical Sciences, Shiraz, Iran. Email: m2620.shaygan@gmail.com Shiraz University of Medical Sciences Shiraz Iran m2620.shaygan@gmail.com; 4 Nurse, Ph.D. University of the West of Scotland, Paisley, Scotland. Email: Brian.Thomson@uws.ac.uk University of the West of Scotland University of the West of Scotland Paisley United Kingdom Brian.Thomson@uws.ac.uk

**Keywords:** students, nursing, nurses, male, curriculum., estudiantes de enfermería, enfermeros, curriculum., estudantes de enfermagem, enfermeiros, currículo.

## Abstract

**Objective.:**

The present study aimed to determine male nursing students’ perception of gender barriers in nursing curricula.

**Methods.:**

This descriptive study was conducted on 150 B.Sc. and M.Sc. nursing students at Tabriz School of Nursing and Midwifery, Tabriz university of medical sciences, Tabriz, Iran that were selected through convenience sampling. The study data were collected using Inventory of Male Friendliness in Nursing Programs-Short (IMFNP-S). This scale has 17 items for investigating male nursing students’ perception of gender barriers in nursing curricula. Each item is a 5-point Likert-type scale scored from 0 to 4; total scale score could range from 0 to 68, higher scores representing male nursing students’ perception of less gender barriers in nursing curricula.

**Results.:**

The total mean score of gender barriers was 35.11+6.15. The most important barriers included different requirements/limitations in obstetrics apprenticeship (Median=1), and need for proving oneself because of people’s expectation of nurses to be female (Median=2). On the other hand, the least important barriers were lack of important people’s support on one’s career decisions (Median=3), and lack of opportunity to work with other male nurses (Median=3). The scale score was not associated with the socio-demographic characteristics studied.

**Conclusion.:**

The most male nursing students feel various gender issues in the nursing curriculum in a medium level that may negatively impact on their learning, professional performance and motivation and tendency to nursing. Furthermore, this vicious cycle can lead to lack of professional development, leaving the job and burnout. Thus, creating a gender-neutral environment can make nursing programs more male friendly.

## Resumen

**Objetivo.:**

Describir la percepción de los estudiantes varones de enfermería sobre las barreras de género en los planes de estudio de enfermería.

**Métodos.:**

Este estudio descriptivo se llevó a cabo con la participación de 150 varones estudiantes de enfermería de la Escuela de Enfermería y Partería de Tabriz, Universidad de Ciencias Médicas de Tabriz, Irán, seleccionados mediante un muestreo por conveniencia. Los datos del estudio se recogieron utilizando el Inventory of Male Friendliness in Nursing Programs-Short (IMFNP-S). Esta escala tiene 17 ítems con opciones de respuesta tipo Likert de 5 puntos que se puntúa de 0 a 4; la puntuación total de la escala puede oscilar entre 0 y 68, y las puntuaciones más altas representan la percepción de menos barreras de género.

**Resultados.:**

La puntuación media total de las barreras de género fue de 35.11+6.15. Las barreras más importantes incluían los diferentes requisitos o limitaciones en el aprendizaje de la obstetricia (Mediana=1), y la necesidad de probarse a sí mismo debido a la expectativa de la gente de que las enfermeras sean mujeres (Mediana=2). Por otro lado, las barreras menos importantes fueron la falta de apoyo de personas importantes en las decisiones de la carrera profesional (mediana=3) y la falta de oportunidades para trabajar con otros enfermeros (mediana=3). La puntuación de la escala no se asoció con las características sociodemográficas estudiadas.

**Conclusión.:**

La mayoría de los estudiantes varones de enfermería sienten diversas barreras de género en el plan de estudios de enfermería en un nivel medio, que puede repercutir negativamente en su aprendizaje, motivación, rendimiento profesional y en la tendencia hacia la enfermería. Además, esta situación puede conducir a la falta de desarrollo profesional, al abandono del trabajo y al agotamiento. Así pues, la creación de un entorno neutral desde el punto de vista de género puede hacer que los programas de enfermería sean más favorables a los hombres.

**Objetivo.:**

Descrever a percepção dos estudantes de enfermagem homens sobre as barreiras de gênero nos planos de estudo de enfermagem.

**Métodos.:**

Este estudo descritivo se levou a cabo com a participação de 150 homens que eram estudantes de enfermagem da Escola de Enfermagem e Parteira de Tabriz, Universidade de Ciências Médicas de Tabriz, Irã, que foram selecionados mediante uma amostragem por conveniência. Os dados do estudo se recolheram utilizando o Inventory of Male Friendliness in Nursing Programs-Short (IMFNP-S). Esta escala tem 17 itens com opções de resposta tipo Likert de 5 pontos que se pontua de 0 a 4; a pontuação total da escala pode oscilar entre 0 e 68, e as pontuações maiores representam a percepção de menos barreiras de gênero.

**Resultados.:**

A pontuação média total das barreiras de género foi de 35.11+6.15. As barreiras mais importantes incluíam os diferentes requisitos ou limitações na aprendizagem da obstetrícia (Média=1), e a necessidade de provar-se a si mesmo devido à expectativa das pessoas de que as enfermeiras sejam mulheres (Média=2). Por outro lado, as barreiras menos importantes foram a falta de apoio de pessoas importantes nas decisões da carreira profissional (média=3) e a falta de oportunidades para trabalhar com outros enfermeiros (média=3). A pontuação da escala não se associou com as características sociodemográficas estudadas.

**Conclusão.:**

A maioria dos estudantes de enfermagem homens sentem diversas barreiras de gênero no plano de estudos de enfermagem num nível médio, que pode repercutir negativamente na sua aprendizagem, motivação e rendimento profissional, e tendência à enfermagem. Além disso, esta situação pode conduzir à falta de desenvolvimento profissional, ao abandono do trabalho e ao esgotamento. Assim pois, a criação de um entorno neutral desde o ponto de vista de gênero, pode fazer que os programas de enfermagem sejam mais favoráveis aos homens.

## Introduction

Nurses, as the most important part of the professional force of healthcare systems, are at the frontline of service provision and possess various roles and responsibilities.(1) Therefore, any shortcoming in this group has a direct impact on the quality and quantity of healthcare services and, consequently, the health of individuals and society.(1) Review of the literature indicates that men’s presence is crucial in nursing profession.(2) According to a recent research, the number of men pursuing a Bachelor of Nursing degree is slightly more than 10%. Males make up just 9.9% and 6.8% of postgraduate nursing degrees, respectively, such as Master of Science Nursing and Doctoral-Level Nursing degrees.(3) According to the Ministry of Health and Medical Education (MOHME), in Iran women make up roughly 75% of the nursing workforce, while males make up 25%.(4)

Men’s role in nursing profession has been neglected.(5) Men are also aware of this fact, but are not highly interested in this regard.(5) This might have resulted from healthcare personnel’s gender-based stereotype and inappropriate viewpoint towards men’s presence in this profession(5) and it might also be attributed to each region’s cultural and social conditions. In other words, men’s presence and attitude towards the nursing profession can be different depending on society’s perspective, culture, and traditions.(6) These factors affect the role of the nursing profession in society’s health and patient care, and give this profession some dimensions that make the negligence of a gender-based perspective undeniable.(7) So, there is a need to develop strategies to attract men to this profession. On the other hand, men are at a higher risk of resignation from nursing programs.(8) Resignation from nursing programs would have an emotional cost, as well, because nursing students would feel ashamed and emotionally exhausted after resigning from nursing program.(9)

Although years have passed since men’s presence in the nursing profession, they do not play an effective role in this respect.(5) Men’s employment and survival in nursing has turned into a challenge resulting from various educational as well as social barriers.(10) Therefore, with the expansion of nursing programs, men’s needs in this profession should be determined.(11) Understanding the barriers that lead to resignation from nursing programs in high-risk populations might be effective in reducing the rate of attrition in this profession.(11) The majority of the research were qualitative (context-based) and focused only on the perceptions of male student nurses about the nursing profession.(12) There are limited studies about the perceptions of male nursing students about gender barriers in nursing curricula. It is quite evident that gender has overshadowed all dimensions of nursing and inattention to this factor could lead to negligence of its impacts on the profession, eventually endangering its survival.(13) There is a need to explore the perceptions of nursing students about nursing barriers in curricula that men are facing. Therefore, the present study aims to determine male nursing students’ perception of gender barriers in their nursing curricula.

## Methods

Design of the study. This descriptive study aimed to determine male nursing students’ perception of gender barriers in nursing curricula at Tabriz University of Medical Sciences in 2020.

Participants. The research population included all male B.Sc. nursing students at the end of the second year to the fourth year of education (*n*=128) and all male M.Sc. nursing students at the end of the first and second year of education (*n*=22) in Tabriz School of Nursing and Midwifery. The study sample size was determined based on a pilot study. The participants included 150 nursing students selected through convenience sampling. The inclusion criteria of the study were being willing to take part in the research, being a B.Sc. or M.Sc. student, being a student at Tabriz University of Medical Sciences, and not being a guest or transfer student.

Instrument. In order to determine the male nursing students’ perception of gender barriers in nursing curricula, Inventory of Male Friendliness in Nursing Programs-Short (IMFNP-S) (14) was used. This inventory included demographic information (12 items) and some questions for investigating male nursing students’ perception of gender barriers in nursing curricula (17 items). The items could be responded through a 5-point Likert scale with the following options: completely agree, agree, no idea, disagree, and completely disagree. In order to avoid bias, some items were scored reversely. Overall, the items scores could range from 0 to 4 (0: completely disagree and 4: completely agree in positive sentences; 0: completely agree and 4: completely disagree in reverse ones). Accordingly, 0 and 4 represented dominant presence and lack of barriers, respectively. Besides, the scores could range from 0 to 68 with higher scores representing male nursing students’ perception of less gender barriers in nursing curricula. This questionnaire was developed by Chad Elis O’Lynn based on a strong review of the literature in 2004. The validity of the questionnaire was approved using nursing experts’ opinions. Its reliability was also confirmed by Cronbach’s alpha = 0.84.(14)

Ethics. After gaining the approval of the University’s Research Council and obtaining ethical approval, the questionnaires were distributed. At first, the participants were explained about the research and its voluntary nature and were reassured about the secrecy and confidentiality of their information. Then, they were invited to take part in the study.

Procedure. After receiving oral and written explanations at the beginning of the questionnaire, they completed the questionnaires through self-report. Data collection was done within two months in 2020.

Data analysis. After all, the data were entered into the SPSS statistical software, version 16. The descriptive variables were analyzed using descriptive (frequency, mean, Standard Deviation -SD). The mean and SD were calculated for the total scale score and for each item the median, minimum, and maximum values were calculated according to Sullivan and Artino's recommendation for the interpretation of Likert-type scale data.(18) For inferential analysis, Kruskal-Wallis and Mann-Whitney tests and Pearson’s correlation coefficient were used.

## Results

The study participants’ demographic characteristics have been presented in Table 1. The mean total score of gender barriers was 35.11±6.15. Based on the study results, five main educational barriers included different requirements/limitations in obstetrics (OB) apprenticeship, need for proving oneself because of people’s expectation of nurses to be female, faculty’s referral to nurses using feminine prepositions, different treatments against male and female nursing students, and getting nervous after being accused of sexual inappropriateness after touching female patients. On the other hand, the least important barriers were lack of important people’s support on one’s career decisions, lack of opportunity to work with other male nurses, and lack of content on different communication styles (Table 2).

**Table 1:** The study participants’ socio-demographic characteristics

Socio-demographic variables	
Age; Mean±SD	23.97±4.29
Marital status; *n* (%)	
Single	126 (87.5)
Married	18 (12.5
Semester; n (%)	
3	10 (7)
4	64 (45.1)
5	6 (4.2)
6	32 (22.5)
8	30 (21.1)
Academic degree; *n* (%)	
Bachelor of science	128 (85.3)
Master of science	22 (14.7)
Grade point Average; Mean±SD	15.57±1.36
Family’s economic status; *n* (%)	
Income equal to expenses	98 (68.1)
Income less than expenses	20 (13.9)
Income over expenses	26 (18.1
Ethnic group; *n* (%)	
Turk	130 (89)
Persian	6 (4.1)
Kurd	10 (6.8
Existence of nurses among family members; *n* (%)	44 (29.7)
Men’s faculty activity in nursing school; *n* (%)	112 (77.8)
Informal work experience in nursing; *n* (%)	48 (32.4)
With formal work experience in nursing; *n* (%)	20 (13.5)

**Table 2:** Gender-based barriers as reported by the respondents

Median	Max	Min	Items
2	4	0	1. Faculty referred to nurse as “she”
2	4	0	2. No history of men in nursing
2	4	0	3. No active recruitment of men
2	4	0	4. Faculty made disparaging remarks about men
2	4	0	5. No content on men’s health
3	4	0	6. No opportunity to work with male RNs
1	4	0	7. Different requirements/limitations in OB
2	4	0	8. No content on different communication styles
2	4	0	9. Not being invited to participate in all student activities
2	4	0	10. Not being encouraged to strive for leadership roles
3	4	0	11. People important to me did not support my career decision
2	4	0	12. I felt I had to prove myself because people expected nurses to be female
2	4	0	13. Male and female students were treated differently
2	4	0	14. Gender was a barrier in developing collegial relationship/faculty
2	4	0	15. I did not feel welcomed by staff RNs
2	4	0	16. I was nervous when a woman would accuse me of sexual inappropriateness when I touched her
3	4	0	17. My nursing program did not prepare me to work with females
2	3	1	Total score on Likert scale
34	51	14	Total score

The results revealed no significant relationships between the students’ socio-demographic features and their total score of gender barriers (Table 3).

**Table 3:** Relationships between the students’ socio-demographic variables and their gender barriers

p-value	Statistical tests	Mean±SD	n	Variables
0.42	r= 0.09	23.97±4.29	142	Age
Marital status				
Single	126	35.28±5.65	0.91	U=277
Married	18	35.78±5.99		
Semester			*x*2=7.96	0.09
3	10	32.40±1.14	Df=4	
4	64	36.81±6.28		
5	6	28.33±1.03		
6	32	33.81±3.88		
8	30	36.73±4		
Academic degree			0.49	U=306
Bachelor of science	128	35.11±6.35		
Master of science	22	35.09±5.07		
Family’s economic status			*x*2=3.71	0.16
Income equal to expenses	98	35.98±5.74	Df=2	
Income less than expenses	20	31.60±5.98		
Income over expenses	26	34.77±7.08		
Ethnic group			*x*2=1.50	0.47
Turk	130	35±6.10	Df=2	
Persian	6	32±2.64		
Kurd	10	37.60±8.08		
Existence of nurses among family members			U=534	0.65
Yes	44	35.50±3.89		
No	104	35.02±6.95		
Men’s faculty activity in nursing school			U=534	0.4
Yes	112	35.23±5.81		
No	32	35.19±7.79		
Informal work experience in nursing			U=591.50	0.4
Yes	48	35.17±6.62		
No	100	35.16±6.01		
Formal work experience in nursing			U=296	0.70
Yes	20	37±6.72		
No	128	34.87±6.09		

## Discussion

The study findings showed that the male nursing students’ mean score of gender barriers was 35.11+6.15. In addition, five main educational barriers included different requirements/limitations in OB apprenticeship (Median=1), need for proving oneself because of people’s expectation of nurses to be female (Median=2), faculty’s referral to nurses using feminine prepositions (Median=2), different treatments against male and female nursing students (Median=2), and getting nervous after being accused of sexual inappropriateness after touching female patients (Median=2).

In the study performed by Spahr,(16) the mean score of gender barriers was 56.47+9.42. In this study, the students’ scores were lower compared to similar studies, which indicates that our participants were faced with more gender barriers. It seems that differences in research settings and students’ educational and cultural backgrounds might affect their perceptions of gender barriers. In this context, further researches are required to eliminate gender barriers and improve nursing curricula for male nursing students in Iran.

Experience of working in an OB apprenticeship and fear of being accused of sexual inappropriateness have been mentioned by participants in the present study and other studies conducted on the issue.(17,18) This can lead to considerable stress.(18) Generally, differences/limitations in OB apprenticeship and getting nervous after being accused of sexual inappropriateness are inter-related. Working with women at the time of delivery requires a close and intimate relationship with nurses. Since all such patients are female, OB apprenticeship can be a challenging area for male nurses.(17,18) In fact, the OB apprenticeship is a place where students feel role conflicts.(18) It might be attributed to the fact that female nurses are patients’ first priority, particularly in case they require intimate care. This barrier results in a reduction of the complete experience of what must be presented to male nursing students. In other words, male students would have unequal learning in an OB apprenticeship.(19) Of course, limitations in the learning environment are not only affected by patients’ lack of interest in having male nurses, but also by nursing instructors’ and personnel’s lack of tendency to present such care in hospitals.(20) Providing female patients with intimate care is the main factor during the OB apprenticeship.(21) Although touching is a main component of nursing care, it is often considered to be a source of anxiety for male nurses because touching is a feminine feature and might be interpreted as a sexual act when performed by men.(17,18) Thus, educational programs regarding how to use touch are highly essential for male nursing students. In this respect, colleges can help make male nursing students close to patients and their families. It is also necessary to improve communication skills to increase male nurses’ compatibility with patients and their spouses.(22) In this regard, informing society about men’s position in the nursing profession can be beneficial and decrease male students’ tensions in OB apprenticeship.

In the present study, the need to prove oneself because of people’s expectations of nurses to be female was one of the most important educational barriers reported by nursing students. This might be attributed to the feminine viewpoint towards the nursing profession(23) as well as higher expectations from male nursing students and graduates in society.(24) One of the main educational barriers for male nursing students was faculty’s referral to nurses using feminine prepositions. In other words, nursing instructors are perpetuating the gender stereotype in the nursing profession. Powers *et al.*(25) reported findings about narrow focused nursing programs and that they are learned through a feminine viewpoint. Some nursing schools used a feminine pedagogy because they were afraid of destroying unique nursing qualities.(26) The results also proved that nursing programs continued to stabilize gender-based stereotypes through feminization of the curricula, which could eventually lead to gender dissonance and male students’ resentment.(26). Furthermore, evidence has indicated that nurses are still referred to by feminine prepositions(25) and some educational subjects are sexually biased.(26)

As mentioned above, faculty’s referral to nurses using feminine prepositions was one of the main gender barriers in the current study. Although changing old verbal patterns might be difficult, it is of particular importance for male nursing students. Similar to other communities, Iranian society has challenged many gender-stereotyped titles over time. This change is also possible in the nursing profession similar to other gender-specific professions. In this context, cultural and social infrastructures for eliminating gender-based views towards the nursing profession are needed to modify instructors’ verbal patterns and nursing curricula.

Different treatments against male and female nursing students can be attributed to feminine pedagogy, gender-based bias in the nursing profession, men’s practical limitations (e.g. in OB wards), and nursing instructors’ thoughts and perspectives. Male nursing students and clinical nurses experienced tokenism and social isolation.(27) On the other hand, Mao *et al.* showed that Males can turn barriers into facilitators and there is no clear line between gender-related advantages and disadvantages as factors impacting professional development.(28) It seems that the creation of infrastructure regarding men’s position in the nursing profession, providing authorities with the necessary training, and making society members aware of men’s vital role in the nursing profession can play a role in eliminating this barrier. By addressing social stereotypes of femininity and masculinity and promoting the concept of gender neutrality, nursing organizations and nursing education programs can help with recruiting. In order to counteract gender bias and recruit more male students, nursing schools must be more active in their efforts.

In the present study, the least important barriers included lack of important people’s support on one’s career decisions (Median=3), lack of opportunity to work with other male nurses (Median=3), and lack of content on different communication styles (Median=2). Similarly, Davidson(29) and Spahr(16) mentioned the lack of important people’s support for one’s career decisions as one of the least important gender barriers. In the previous decades, parents’ pressure was a barrier against men’s admission to the nursing profession.(30) Fortunately, this barrier has been eliminated to a great extent nowadays. It seems that the labor market and prosperity have been effective in changing families’ opinions in this regard. Considering lack of opportunity to work with other male nurses, this opportunity has enhanced by increase in the population of male nursing students and graduates.

In the current study, the students did not consider lack of content on different communication styles to be an important barrier. This might be due to the fact that receiving necessary training regarding communication styles is not highly essential in the nursing profession. The students’ unawareness of the importance of communication styles in care provision could play a role, as well.

Limitations and Suggestions. This study was performed on male nursing students in Tabriz University of Medical Sciences. Therefore, the results cannot be generalized to other nursing schools and further studies are needed to determine gender barriers in other nursing schools in different parts of the country. Additionally, this study investigated gender barriers from male students’ perspectives. Thus, future studies are recommended to assess nursing faculty’s perception in this regard and compare their viewpoints to those of nursing students. Moreover, using observers for judging classes and videotaping classes are suggested as instruments for assessment of sexual and ethnic biases. Finally, the results of the present study were based on the participants’ ability to understand questions clearly and remember what had occurred during their training. Although using previously-designed reliable instruments can decrease misinterpretation of questions, further studies are recommended to develop more up-to-date instruments specifically for Iranian culture.

## Conclusion

Because of the importance of men’s presence in the nursing profession, nursing curricula have to be improved by the elimination of gender barriers for male nursing students. So, in this study, male nursing students’ perception of gender barriers in nursing curricula at an Iranian University of Medical Sciences was assessed. The male nursing students’ mean score of gender barriers was at a medium level. It shows that most male nursing students feel various gender issues in the nursing curriculum at an average level. This level of gender barriers may have a negative impact on their nursing learning, professional performance, motivation, and tendencies. Furthermore, this vicious cycle can lead to a lack of professional development, leaving the job, fault, and burnout.

Some of the most common gender barriers identified in this study, such as different requirements/limitations in OB apprenticeship, need for proving oneself because of people’s expectation of nurses to be female, faculty’s referral to nurses using feminine prepositions, different treatments against male and female nursing students, and getting nervous after being accused of sexual inappropriateness after touching female patients, can be a source of stress and tension for male nursing students.

Since male gender can be a risk factor for resignation from nursing programs, these programs should consider the reasons for male nursing students’ resignation. Besides, nursing instructors who are mainly female should be aware of their effective role in the creation of a neutral, male-friendly environment. In this regard, being sensitive to students’ needs can help male students feel accepted and supported. Providing male students with training about how to face gender barriers can be influential, as well. Making some changes in nursing curricula and the general atmosphere of nursing school can also direct nursing programs towards male friendliness. In fact, the creation of a sexually neutral environment is a necessity for male students’ success in such a female-dominant profession. Training nursing personnel in hospitals regarding the elimination of sexual discrimination can help reduce this barrier for male nursing students. Moreover, further studies have to be conducted on gender bias in nursing education and the impacts of this bias on men’s employment and survival in the nursing profession.
